# Reviewers for 2020

**DOI:** 10.4314/gmj.v54i4.1

**Published:** 2020-12

**Authors:** David Ofori-Adjei

**Affiliations:** Editor-in-Chief, Ghana Medical Journal editor@ghanamedj.org

Reviewers contribute greatly to shaping the quality of manuscripts published in our journal. Our reviewers give of their time and expertise to the service of science. We recognise those who reviewed for us in 2020. We hope that many more will respond to our invitation in the coming years.

**Table:** 

NAME	COUNTRY
Abang, Innocent	Nigeria
Abantanga, Francis	Ghana
Abdulai, Alhassan	Ghana
Abraham, Emem	Nigeria
Addo-Lartey, Adolphina	Ghana
Addo, Edwina Beryl	Ghana
Addo, Nii Akwei	Ghana
Adedia, David	Ghana
Adegbehingbe, Olayinka	Nigeria
Adeko, Oluseun	Nigeria
Adeniran, Abiodun	Nigeria
Adeniyi, Oluwafunmilayo	Nigeria
Adenusi, Adedotun	Nigeria
Adisa, Rasaq	Nigeria
Adjei, George	Ghana
Adjei, Patrick	Ghana
Adu-Ababio, Francis	Ghana
Adu, Emmanuel	Ghana
Afrane, Adwoa	Ghana
Afriyie-Mensah, Jane	Ghana
Agbeno, Evans	Ghana
Agongo, Erasmus	Ghana
Agyei-Nkansah, Adwoa	Ghana
Agyeman-Yeboah, Joana	Ghana
Aiyekomogbon, Joshua	Nigeria
Ajayi, Akinola	Nigeria
Aje, Akinniyi	Nigeria
Akakpo, Patrick	Ghana
Akase, Iorhen	Nigeria
Akinmoladun, Janet	Nigeria
Akodu, Babatunde	Nigeria
Akpalu, Albert	Ghana
Akpalu, Josephine	Ghana
Amedonu, Edem	Ghana
Amoakoh-Coleman, Mary	Ghana
Amoaku, Winfried	UK
Amofah, George	Ghana
Amoyaw-Asamoah, Isabella	Ghana
Anane-Fenin, Betty	Ghana
Anim, Jehoram	Ghana
Aniteye, Ernest	Ghana
Ankobiah, William	Ghana
Ansah, Evelyn	Ghana
Ansah, Justina	Ghana
Antwi, Sampson	Ghana
Anyanechi, Charles	Nigeria
Aryeetey, Richmond	Ghana
Asaleye, Christianah	Nigeria
Asamoah, Kojo	Ghana
Asante, Ivy	Ghana
Asare Quansah, Gloria	Ghana
Asiamah, Samuel	Ghana
Asuquo, Marcus	Nigeria
Atiase, Yacoba	Ghana
Atta, Dzifa	Ghana
Attoh, Seth	Ghana
Avancini, Joao	Brazil
Awosan, Kehinde	Nigeria
Awuku, Mark	Canada
Awuku, Yaw	Ghana
Ayinmode, Adekunle	Nigeria
Ballal, Arjun	India
Bandoh, Delia	Ghana
Bediako-Bowan, Antoinette	Ghana
Behera, Prateek	India
Bello, Ajediran	Ghana
Benfor, Bright	Morocco
Biritwum, Richard	Ghana
Boakye, Hosea	Ghana
Boima, Vincent	Ghana
Bojuwoye, Matthew	Nigeria
Bonavina, Luigi	Italy
Bonney, Joseph	Ghana
Braimah, Ramat	Nigeria
Bruce, Ishmael	Canada
Calys-Tagoe, Benedict	Ghana
Clegg-Lamptey, Joe Nat	Ghana
da-Costa Vroom, Frances	Ghana
Dakubo, Jonathan	Ghana
Dame, Joycelyn	Ghana
Darteh, Eugene	Ghana
De Souza, Dziedzom	Ghana
Dei-Adomakoh, Yvonne	Ghana
Der, Edmund	Ghana
Desalu, Ibironke	Nigeria
Donkor, Peter	Ghana
Edwin, Frank	Ghana
Edzie, Emmanuel	Ghana
Egyir, Beverly	Ghana
Ekem, Ivy	Ghana
Enimil, Anthony	Ghana
Fasina, Oluyemi	Nigeria
Fiakpornoo, Martina	Ghana
Forson, Audrey	Ghana
Gezawa, Ibrahim	Nigeria
Gupta, Nishkarsh	India
Hodgson, Abraham	Ghana
Holdbrook-Smith, Henry	Ghana
Hviid, Lars	Denmark
Idowu, Bukunmi	Nigeria
Issah, Kofi	Ghana
Kenu, Ernest	Ghana
Khalifa, Ahmed	Egypt
Kirfi, Abdullahi Musa	Nigeria
Kluivers, K.B.	Netherlands
Koduah, Augustina	Ghana
Kokuro, Collins	Ghana
Koram, Kwadwo	Ghana
Kretchy, Irene	Ghana
Kwakyi, Edward	Ghana
Kyei, George	Ghana
Kyei, Mathew	Ghana
Lartey, Margaret	Ghana
Lawson, Henry	Ghana
Malm, Keziah	Ghana
Mends, David	Ghana
Mensah, samuel	Ghana
Mensah, Yaw	Ghana
Merga, Hailu	Ghana
Michael, Godpower	Nigeria
Mintah, Felix	Ghana
Mock, Charles	USA
Mohd Noor, Noor Haslina	Malaysia
Morhe, Emmanuel	Ghana
Mould-Millman, Nee-Kofi	USA
Mula, Marco	UK
Mume, Celestine	Nigeria
Munga, Judith	Kenya
Mustapha, Shettima	Nigeria
Nartey, Stella	Ghana
Newman, Mercy	Ghana
Nguah, Samuel	Ghana
NkyeKyer, Kobina	Ghana
Nottidge, Bolanle	Nigeria
Nottidge, Timothy	Nigeria
Nsaful, Josephine	Ghana
Nsaful, Kwesi	Ghana
Nuertey, Benjamin	Ghana
Nwaneri, Damian	Nigeria
Nyaaba, Gertrude	Netherlands
Obiegbu, Obinna	Nigeria
Oboirien, Muhammad	Nigeria
Ocran, Benedict	Ghana
Ofori-Adjei, David	Ghana
Ogun, Olufunmilola	Nigeria
Ogun, Olufunmilola	Nigeria
Ogundare, Ezra	Nigeria
Ogunnmodede, Adebusola	Nigeria
Ohene-Yeboah, Michael	Ghana
Oishi, Akio	Japan
Okonkwo, Ogugua	Nigeria
Olagunju, Andrew T.	Canada
Olamoyegun, Michael	Nigeria
Olaseni, Abayomi	Nigeria
Olasode, Olayinka	Nigeria
Oluyemi, Aderemi	Nigeria
Omidiji, Olubukola	Nigeria
Onyango, Dickens	Kenya
Opare, Joseph	Ghana
Oppong, Samuel	Ghana
Osei-Ampofo, Maxwell	Ghana
Osman, Kwabena	Ghana
Ossai, Edmund Ndudi	Nigeria
Osunde, Otasowie	Nigeria
Owusu-Antwi, Ruth	Ghana
Owusu, Enid	Ghana
Oyewole, Olufemi	Nigeria
Paintsil, Elijah	USA
Puplampu, Peter	Ghana
Quarde, Akuffo	USA
Rahman, Ganiyu	Saudi Arabia
Renner, Lorna	Ghana
Rockwell, William	United States
Sabblah, George	Ghana
Sagoe, Kwamena	Ghana
Samaila, Modupeola	Nigeria
Sarfo, Fred	Ghana
Searyoh, Kafui	Ghana
Seidu, Abdul-Aziz	Ghana
Shittu, Akeem	Nigeria
Suleiman, Zakari	Nigeria
Sumankuuro, Joshua	Australia
Sylverken, Augustina	Ghana
Tachi, Kenneth	Ghana
Tadakamadla, Santosh	Australia
Tagoe, Lily	Ghana
Tampuori, John	Ghana
Technau, Karl-Gunter	South Africa
Tenkorang, Eric	Ghana
Tetteh, Raymond	Ghana
Torpey, Kwame	Ghana
Ubah, Josephine	Nigeria
Udofia, Emilia	Ghana
Ukegbu, Patricia	Nigeria
Welbeck, Jennifer	Ghana
Wu, Lily	Ghana
Yang, Fan	China
Yawson, Alfred	Ghana
Yeboah-Manu, Dorothy	Ghana
Yorke, Ernest	Ghana
Zaman, Tabrez	Saudi Arabia

